# How do risks and benefits affect user’ privacy decisions? An event-related potential study on privacy calculus process

**DOI:** 10.3389/fpsyg.2023.1052782

**Published:** 2023-02-16

**Authors:** Jialin Fu, Jiaming Zhang, Xihang Li

**Affiliations:** College of Economics and Management, Zhengzhou University of Light Industry, Zhengzhou, Henan, China

**Keywords:** privacy paradox, privacy calculus, event-related potential, decision-making process, app

## Abstract

**Purpose:**

The purpose of this study is to examine how risks and benefits affect users’ privacy-related decision-making processes.

**Design/methods/approach:**

This study collected and analyzed the neural activity processes of users’ privacy-related decisions when faced with personalized services with different risks and benefits through an ERP experiment that included 40 participants.

**Findings/results:**

The findings show that users subconsciously categorize personalized services based on benefit; Privacy calculus affects privacy decision by influencing the allocation of cognitive resources for personalized service, and the scarcity of cognitive resources increases the degree of privacy disclosure; Emotional change in privacy decision is the result of many factors, not the result of privacy risk alone.

**Originality/Discussion:**

This study provides a new perspective to explain the process of privacy decision-making, and a new approach to investigate the privacy paradox.

## Introduction

The rapid development of mobile Internet has brought about a surge in user information, companies are increasingly collecting and storing consumers’ personal data in exchange for a number of benefits (Plangger and Montecchi, 2020). To promote user experience and engagement, many apps have developed personalized services. Personalized services, as an important product of digital economy, recommend information or products that may be of interest to users based on their browsing history or preferences and behaviors of similar users (Xu et al., 2011; Li et al., 2017; Lee and Yuan, 2020). However, the negligence of many enterprises in managing user privacy has led to a series of privacy leakage and privacy trafficking incidents. For example, after Cambridge Analytica violated Facebook’s terms of service by stealing the data of more than 80 million users for use in supporting U.S. President Donald Trump during the 2016 presidential election. Also in 2021, the privacy leakage occurred again when users of a hacker forum published hundreds of millions of Facebook user data, including phone numbers and other personal information, for free online. Privacy leaks abounded in China when 8 million registered users’ information was leaked from the Xiaomi forum and over 70 million QQ group data was leaked from Tencent. These privacy leaks have caused users to concern about the security of personalized services and even aversion to personalized services among most users, making it less effective. Interestingly, despite showing privacy concerns, many users continue to disclose information and use personalized services while some users resist, a phenomenon known as the privacy paradox (Lee and Kwon, 2015; Pentina et al., 2016).

To explain this inconsistency of attitude and behavior, scholars have conducted many studies on privacy paradox. The most commonly used model in privacy research is the privacy calculus model, in which people rationally weigh the benefits and costs carefully when considering whether to disclose personal information; in other words, users’ privacy decisions are made based on the balance between risks and benefits (Anderson and Agarwal, 2011; Xu et al., 2011). When the privacy benefits exceed the privacy costs, users will disclose information and use personalized services; otherwise, they will retain information (Norberg et al., 2007; Anderson and Agarwal, 2011). However, some scholars argue that people are not fully rational and emotional when making decisions (Acquisti et al., 2007; Volz and Gigerenzer, 2012). Some scholars explain people’s fluke mentality that disclosing privacy will not have serious implications and selectively ignore possible risks from the perspective of limited rationality (Li et al., 2015). on the other hand, Social Theory argues that the behavior of individuals is influenced by a variety of social factors such as social relationships, social norms, and social institutions, which make it impossible for individuals to make decisions based solely on themselves (Acquisti and Grossklags, 2004), for example, some users share their privacy in order to gain social opportunities (Agi and Jullien, 2018). Construal Level Theory suggests that people’s interpretation of events changes with their perception of the mental distance to the e’ent, which in turn affects their behavior (Krasnova and Veltri, 2010; Bandara et al., 2017). However, human cognitive ability is limited, users’ perception and usefulness of privacy is not clear in big data environment, privacy is abstract and contextualized to users, and users’ real intention and behavior is difficult to predict (Sordi et al., 2018), and existing scholars still mostly explain the privacy paradox from a concrete cognitive perspective (Li et al., 2018), such as privacy calculus theory measuring gains and losses (Kokolakis, 2017; Wottrich et al., 2018). Although privacy calculus theory has been well studied, we have not known how the risks and benefits affect people’s decision making, which is one of the questions this study will explore.

As mentioned above, users are not completely rational when making privacy decisions, and irrational factors also affect the privacy decision-making process (Acquisti, 2010; Li et al., 2011, 2017; Mohammed and Tejay, 2021), and people are influenced by cognitive biases, habits, intuitions, emotions, etc. before choosing to disclose privacy information. For example, some scholars argue that the “privacy paradox” is due to the existence of cognitive biases such as optimism bias, instant gratification, and illusion of control based on limited rationality (Thaler and Ganser, 2015; Arpetti and Delmastro, 2021). Some scholars argue that people’s disclosure behavior is unconscious (Plangger and Montecchi, 2020), that users do not know the full information about costs and benefits and lack the ability to use the information to make decisions (Gerber et al., 2018), and that habits play an important role before making disclosures (Barth and De Jong, 2017; Fernandes and Pereira, 2021). Tsai et al. (2011) and Brandimarte et al. (2013) suggest that “gut feelings” influence privacy decisions when people feel risky and distant in time and space. In fact, there is a large body of research that demonstrates the importance of emotions in decision-making behavior, Zajonc (1980) suggested that individuals’ emotional responses to stimuli take precedence over cognitive evaluations when making decisions as early as 1980, and anticipated disappointment affect decision outcomes. Loomes and Sugden (1982) and Bell (1982) indicated that expected regret and expected disappointment affect decision outcomes. Sanfey et al. (2003) used FMRI (functional magnetic resonance imaging) techniques to find that unfair allocation schemes triggered emotionally and cognitively relevant brain activity in gamers, and that significant changes in forebrain insula activity in emotionally active brain regions occurred when gamers rejected unfair allocation schemes, thus demonstrating that emotions are related to decision making. In recent years, emotions have also been mentioned in privacy decision making studies, Nyshadham and Castano (2012) indicating that emotions and emotional responses play a secondary role in privacy studies. Scholars have studied emotions in conjunction with perspectives on privacy-related decisions such as intention to disclose information (Anderson and Agarwal, 2011) and risk beliefs (Li et al., 2011). It has been established that positive or negative emotions affect privacy calculations and privacy decisions differently (Anderson and Agarwal, 2011; Li et al., 2011), and that positive emotional responses increase perceived benefits and decrease perceived risks. However, no research has been conducted to experimentally study emotional states in the context of privacy calculus theory, this study hopes to investigate the factors influencing emotional states during privacy decision making in the context of privacy calculus theory.

Previous privacy studies have mostly explored privacy decisions using self-reported survey data such as questionnaires or interviews, but self-reported survey data rely too much on subjects’ subjective perceptions and may contain biased or inaccurate answers (Dimoka, 2011), which cannot shed light on the brain mechanisms underlying complex cognitive process. Brain imaging techniques are the most prevalent tools in neuroscience, and Electroencephalogram (EEG) is widely used to explain individual cognitive process, and its feasibility in consumer behavior research has been fully proved (Telpaz et al., 2015; Barnett and Cerf, 2017). EEG has a high temporal resolution of 1 ms, which allows capturing the macroscopic dynamics of brain activation and synchronization (Alarcao and Fonseca, 2017), corresponding to the phase changes before and after the decision. By using EEG, we can directly measure the neural activity during privacy decision making and look at human “behavior” from the inside in order to provide deeper insights into the relationship between brain and behavior. In this study, based on the privacy calculus theory, event-related potential (ERP) was employed to analyze the neural activity of user privacy related decision-making process. This study experimentally studied how risk and benefit affect uses’ privacy decision-making process, and studied the influencing factors of emotional changes in privacy decision-making process.

## Research hypothesis

Privacy calculus theory is an analytical method based on the cost and benefit trade-offs, which consider the driving and inhibiting factors that affect information disclosure decisions (Anderson and Agarwal, 2011; Xu et al., 2011). In the personalized-privacy decision-making process, users will weigh the potential risk and benefit, which is reflected in the balance of privacy risk and personalized service value (Lee and Kwon, 2015). For enterprises and users, appropriate privacy disclosure is necessary in exchange for personalized services. However, excessive disclosure will bring privacy risks, such as privacy leakage and unwanted advertisement (Pentina et al., 2016). When users believe that benefit of privacy disclosure outweigh the risk, they will disclose their privacy information to obtain personalized service; otherwise, they will retain their privacy (Lee and Kwon, 2015).

Previous studies have found that privacy concern cannot affect users’ privacy disclosure (Taddicken, 2014; Pentina et al., 2016), which means that users with high privacy concern and low privacy concern show no significant different in privacy disclosure, and the factors that really affect users’ privacy decisions are risks and benefits (Xu et al., 2011; Pentina et al., 2016; Hallam and Zanella, 2017; Gruzd and Hernández-García, 2018; Marwick and Hargittai, 2019; Lee and Yuan, 2020). Scholars generally believe that benefits can promote users’ privacy disclosure, while risks inhibit users’ privacy disclosure. However, scholars always disagree on how the benefits and risks affect the decision-making process. Some scholars believe that benefits has a greater impact on users’ privacy disclosure (Lee and Rha, 2016; Zhu et al., 2021), because people trend to give priority to benefit rather than the probability of risk occurrence when making decision (Loewenstein et al., 2001), and risks will only affect users’ intention rather than their actual behaviors (Norberg et al., 2007). One study explains this view from the perspective of construal level theory, pointing out that benefits are concrete and can directly affect behavior, while risks are abstract and can only affect long-term tendencies, i.e., risks are underweighted relative to benefit (Hallam and Zanella, 2017). Other scholars believe that risks have a greater impact on the decision-making process. When facing personalized services, users are always in a state of conflict between risks and benefits (Lee and Rha, 2016), and they will reduce risks through various efforts (Lee and Yuan, 2020).

To investigate how risks and benefits affect user’s privacy decision-making process, P2 and N2 components of ERP components were selected as indicators to analyze the neural process of decision-making. The P2 component, sometimes referred to as P200, is a positive wave that appears about 200 ms after the stimulus is observed and is distributed mainly in the prefrontal and the prefrontal-central association area (Olofsson et al., 2008). It is mainly related to attention and reflects the automatic, rapid and low-level early classification of stimuli. Earlier studies suggested that P2 component reflects the early detection and classification of visual stimuli, a process related to the physical properties of the stimulus (Luck, 2014). The amplitude of P2 component reflects attention bias (Doallo et al., 2007), and the more attention resources are allocated, the greater the amplitude of P2 component is (Noguchi and Murota, 2013). Kahneman and Tversky (2013) introduced reference points into decision making, where the actual outcome of a decision is influenced by the reference point and benefit changes in future. That is when faced with multiple conditions, people will choose one factor as the reference of classification to evaluate value and make decision. In this study, we believe that benefits and risks affect different mental accountings of users, so we propose the following hypothesis:

*H1*: Users classify personalized services by taking benefits as a reference when making personalized privacy decisions, which shows that high value services can induce larger P2 amplitude;

The N2 component is the first negative wave after the P2 component, with a latency of about 200 — 250 ms, distributed mainly in the prefrontal and the prefrontal-central association area (Olofsson et al., 2008). It is related to risk perception and cognitive control, mainly reflecting individual cognitive conflict and risk perception (Yang et al., 2007). The amplitude of N2 component reflects the intensity of cognitive conflict experienced by individuals, and the lager the amplitude, the stronger the cognitive conflict (Eimer, 1993; Yang et al., 2007). People often have various forms of conflict in the cognitive process, such as inconsistency between stimulus and expectation, which will lead to cognitive conflict. In personalized-privacy decisions, cognitive conflict is considered as a trade-off between risks and benefits (Lee and Rha, 2016). Previous research has argued that perceived risks and benefits are independent (Dinev and Hart, 2006; Xu et al., 2009; Li et al., 2011), but recent researches on privacy paradox has found that the psychological perception of risk and benefit is both rational and emotional and interrelated (Mohammed and Tejay, 2021). Therefore, the following hypothesis is proposed:

*H2*: Risk and benefit affect users’ personalized-privacy decision together, which shows that the interaction of risk and benefit significantly affects the amplitude of N2 component.

Decisions are often the result of a combination of rationality and emotion (Dimoka, 2011), as mentioned above, scholars have found that emotion is also an important factor affecting privacy calculus (Anderson and Agarwal, 2011; Li et al., 2011). Earlier, Finucane et al. (2000) showed that high benefit perceptions can increase positive emotions and thus reduce people’s perceptions of risk, and conversely, high risk perceptions can increase negative emotions and lead to lower benefit perceptions. Recently, Li et al. (2017) found that activities of users and websites and the degree of privacy control can trigger positive evaluation and emotions, reduce users’ concerns about privacy risk and increase the possibility of user information disclosure. Mohammed and Tejay (2021) found that in personalized-privacy decision-making, high risk would activate brain areas related to emotional processing and executive function, which leads to allow users to retain privacy information. LPP is an emotion-related ERP component with high temporal sensitivity to emotional arousal, LPP amplitude varies with the intensity of emotional experience during emotion regulation, reflecting the emotional potency of stimulus-induced individuals (Hajcak et al., 2006), and is a good indicator of emotional change (Hajcak and Olvet, 2008). The latency of LPP component is generally ranging within 400–700 ms and it is distributed mainly in the central parietal and posterior areas (Olofsson et al., 2008)，and the midline ERP can be observed around 300 ms after stimulus onset (Hajcak et al., 2009). The LPP is particularly sensitive to emotion regulation in adults (Desatnik et al., 2017), reflecting ongoing fine processing of emotions (Citron, 2012). Previous studies have found that both positive and negative emotions have greater LPP amplitudes than neutral emotions (Hajcak et al., 2006), and stimuli with threat or risk induce larger LPP amplitude than do neutral or positive stimuli (Schupp et al., 2004; Wheaton et al., 2013). Therefore, the following hypothesis is proposed:

*H3*: Risk is the factor that induces emotional changes in the privacy decision-making process, which shows that high risk can induce higher LPP amplitude.

The descriptions of relevant test variables are shown in Table 1.

**Table 1:** Description of relevant test variables.

Variable	Description
Decision-making stage	1 = strongly disagree; 2 = disagree; 3 = not necessarily; 4 = agree; 5 = strongly agree
Privacy risk	The damage that may be caused by the disclosure of the user’s personal information due to illegal or inappropriate use of the information is the user’s expectation of the worst possible outcome
Privacy benefit	Often defined as money or personalized service in return
P2 component	The P2 component is a positive waveform, mainly related to attention, reflecting mainly attentional bias, and the more attentional resources are allocated, the higher the amplitude of the P2 component.
N2 component	The N2 component is the first negative wave after the P2 component, which mainly reflects the individual’s cognitive conflict and risk perception, and the larger the N2 amplitude, the stronger the cognitive conflict.
LPP component	The LPP component is associated with emotion and its amplitude reflects the stimulus-induced emotional potency of the individual.

## Methodology

### Participants

20 participants (9 females, mean age: 22.32), all of whom were from Zhengzhou University of Light Industry, were recruited for this study，the sample size is consistent with most EEG studies (Xu et al., 2011; Seeley et al., 2016; Zotev et al., 2016). They were right-hander, with normal or corrected vision and without any history of neurological or psychiatric illnesses. One male participant was ruled out from the analysis due to excessive artifacts of EEG data, finally leaving 19 data pieces for the data analysis. Before the experiment, the principal investigator explained the experimental precautions to the subject and asked the subject to sign the informed consent form. Participants were paid RMB 70 (about 10 USD) at the end of the experiment.

### Materials

To better simulate the real environment, the study began by selecting the top 30 downloaded apps from the apple store, and then the participants were asked to evaluate the importance of the apps in their daily life through a questionnaire. The top 20% and the bottom 20% were selected as the high-value group and the low-value group, each containing 6 apps. By the degree of privacy protection stipulated in these app privacy protection agreements, app privacy protection can be classified into privacy confidentiality and privacy non-confidentiality. Privacy confidentiality means that the service provider must not applies the users’ privacy information beyond the scopes of the service being provided and of related services, whereas privacy non-confidentiality means that the service provider may share users’ private information with third—party partners in order to provide more accurate recommendations. In this study, privacy confidentiality is defined as low-risk and privacy non-confidentiality as high-risk. Screenshots of personalized recommendation pages of 12 apps were taken as experimental materials. All the experimental materials were adjusted to 720 × 400 with a resolution of 300 pixels in Photoshop.

### Procedure

Before the experiment began, the participants were required to wash their hair with shampoo and blow dry it to keep their scalp clean. Then they entered a dimly lit room and got seated comfortably in reclining chairs. Their eyes were about 1 m away from the computer screen, which was equipped with small keyboard handy for selecting keys.

Before the experiment began, the participants were asked to imagine the following scenario: They had just bought a new mobile phone and installed a series of common apps. The apps provided personalized recommendation services, but required them to agree to the privacy service agreement and provide their privacy information. Anyone who refused to provide such information would be prohibited from enjoying the services in the apps. Everyone then needed to decide whether or not to get the personalized services by providing privacy information based on his/her preferences.

**Figure 1:**
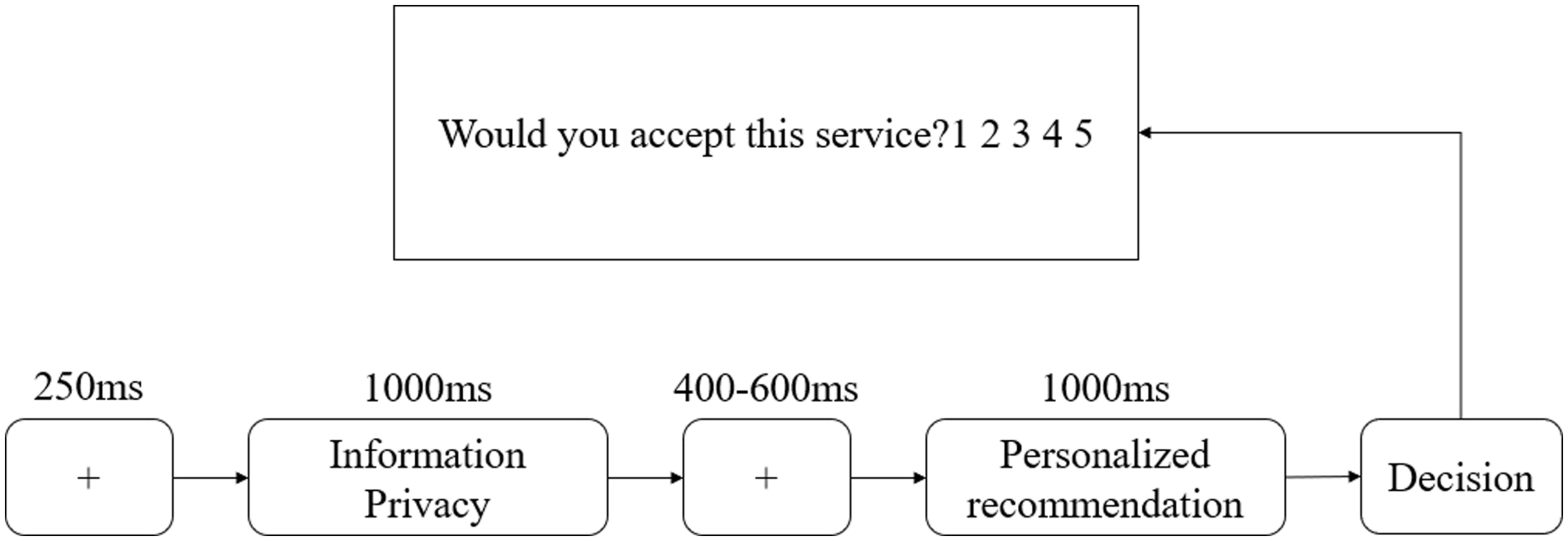
Experimental procedure.

The experiment was divided into two parts, the low-risk part and the high-risk part, and 48 out of a total of 96 trials were randomly presented in each part. In each trial, the participants were first presented with a fixation point lasting 250 ms, followed by a privacy leakage risk level, and a stimulus picture after a delay of 400–600 ms. At the end of the stimulus duration, there was a decision-making stage, in which participants decided at discretion whether or not to provide information. The decision-making stage was based on a five-level scale (1 = strongly disagree; 2 = disagree; 3 = not necessarily; 4 = agree; 5 = strongly agree). The analysis of participants’ behavioral outcomes and whether the experimental results are similar to the expected theoretical results is a prerequisite for the next step in the analysis of how risks and benefits affect privacy decisions. The procedure as shown in Figure 1.

### Data acquisition and preprocessing

A 64-channel NeuSen W64 EEG was used to record the neural data from the participants at a sampling frequency of 1,000 Hz. The electrode positions were in accordance with the international 10–20 system, and E-Prime 3 was used as the stimulus presentation software of the experimental program. During the experiment, the electrode impedance was kept below 10 KΩ.

**Figure 2:**
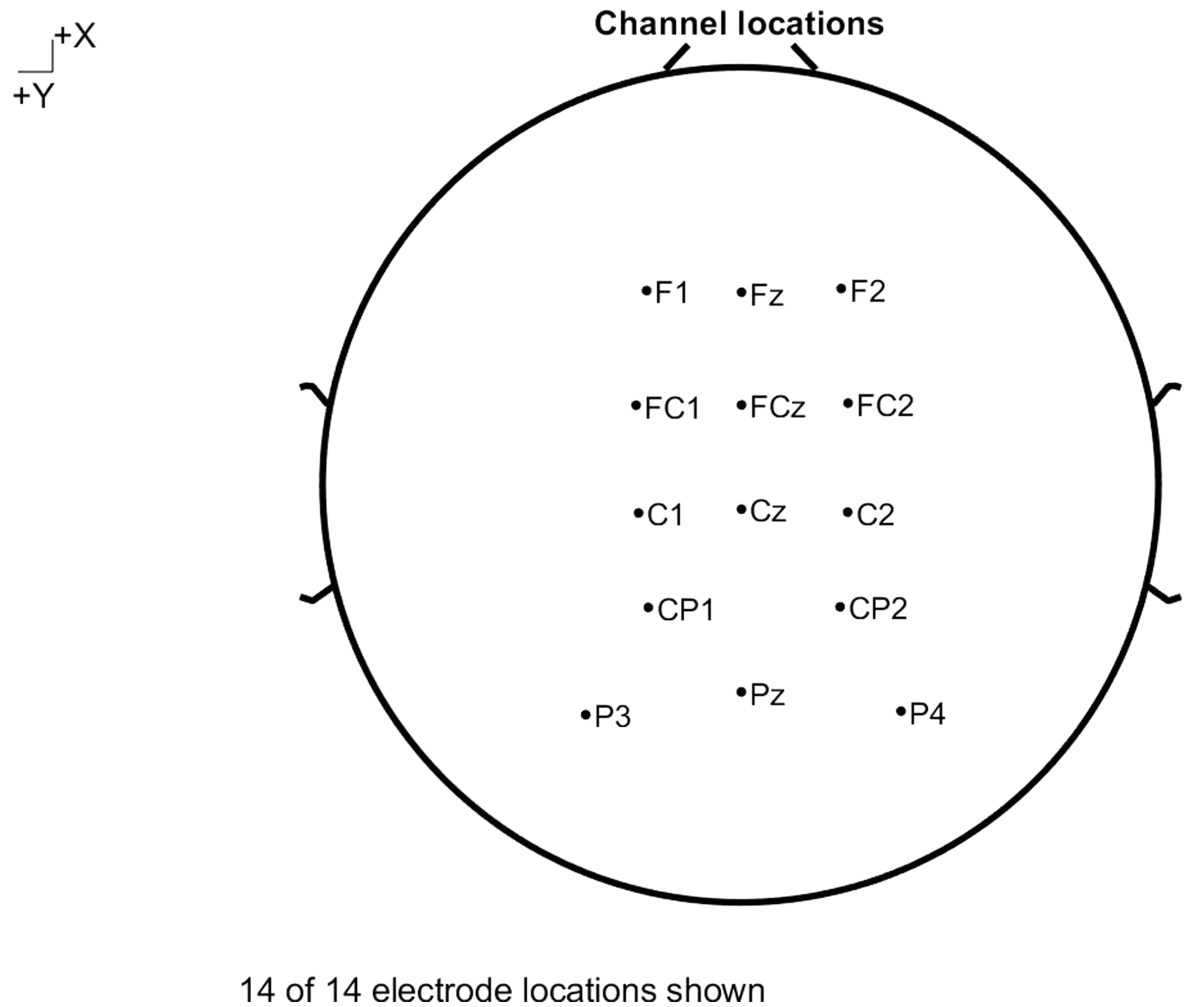
Electrode locations shown.

After the experiment, EEGlab was used to preprocess the original data collected during the experiment. The steps are detailed as follows: ①Lower the sampling frequency from 1,000 to 500 Hz; ②Convert the reference electrode from CPz to the average of all channels; ③Use a 0.01–30 Hz band-pass filter for filtering; ④Segment the data by taking the beginning of picture stimulus as zero point and selecting timespan-200 ms — 800 ms as the time period; ⑤Baseline correction; ⑥Use ICA to remove artifacts; ⑦Superimposed data from a single participant on th average; ⑧Superimposed the data from all participants on the average. Behavioral and ERP data were analyzed using SPSS 20. A total of 14 electrodes involved in the analysis were FZ, F1, F2, FCZ, FC1, FC2, CZ, C1, C2, CP1, CP2, PZ, P3, and P4, as shown in Figure 2. For P2 component, Fz, F1, F2, FCz, FC1 and FC2 were selected as analytical electrodes, and the time window was 170–190 ms in length. The analystical electrodes for N2 component were the same as those for P2 component, expect that the time window was 210–250 ms in length. The analytical electrodes for LPP component were Cz, C1, C2, CP1, CP2, Pz, P3, and P4, with a time window of 550–650 ms. Greenhouse–Geisser correction was performed for data that did not comply with the spherical test.

## Result

### Behavioral results

For acceptance degree of personalized recommendations, a 2 (risks: high-risk vs. low-risk) × 2 (benefits: high-value vs. low-value) repeated measures ANOVA was conducted on participants’ keyboard record. The results show that the main effect of risk is significant, with *F* (1,72) = 21.008, *p* < 0.001, *η^2^* = 0.226; the acceptance degree of personalized recommendation at low risk (*M* = 3.160, SD = 0.608) is significantly higher than that at high risk (*M* = 2.461, SD = 0.743); the main effect of benefit is significant, with *F* (1,72) = 5.044, *p* = 0.028, *η^2^* = 0.065; the degree of acceptance of high-value services (*M* = 2.982, SD = 0.740) is significantly higher than that of low-value services (*M* = 2.640, SD = 0.752); the interaction between them is not significant, with *F* (1,72) = 0.229, *p* = 0.634, *η^2^* = 0.003. The ANOVA result for the behavioral data is shown in Table 2.

**Table 2:** ANOVA results for behavioral data.

Dependent variable	Independent variable	*F*	*P*	*η* ^2^
Decision-making stage	Privacy risk	21.008	0	0.226
	Privacy benefit	5.044	0.028	0.065
	Risk–benefit interaction effect	0.229	0.634	0.003

From the behavior results of participants, we find that privacy risk is negatively correlated with users’ privacy disclosure behavior, while benefit is positively correlated with users’ privacy disclosure behavior. These results are similar to the conclusion of previous studies which investigate the influence of privacy calculus on users’ privacy decision through questionnaires (Marwick and Hargittai, 2019; Lee and Yuan, 2020; Mohammed and Tejay, 2021), indicating that the experiment of this study has been successful, it makes sense to use ERP to analyze how risks and benefits affect privacy decisions for us.

### EEG results

#### P2

**Figure 3:**
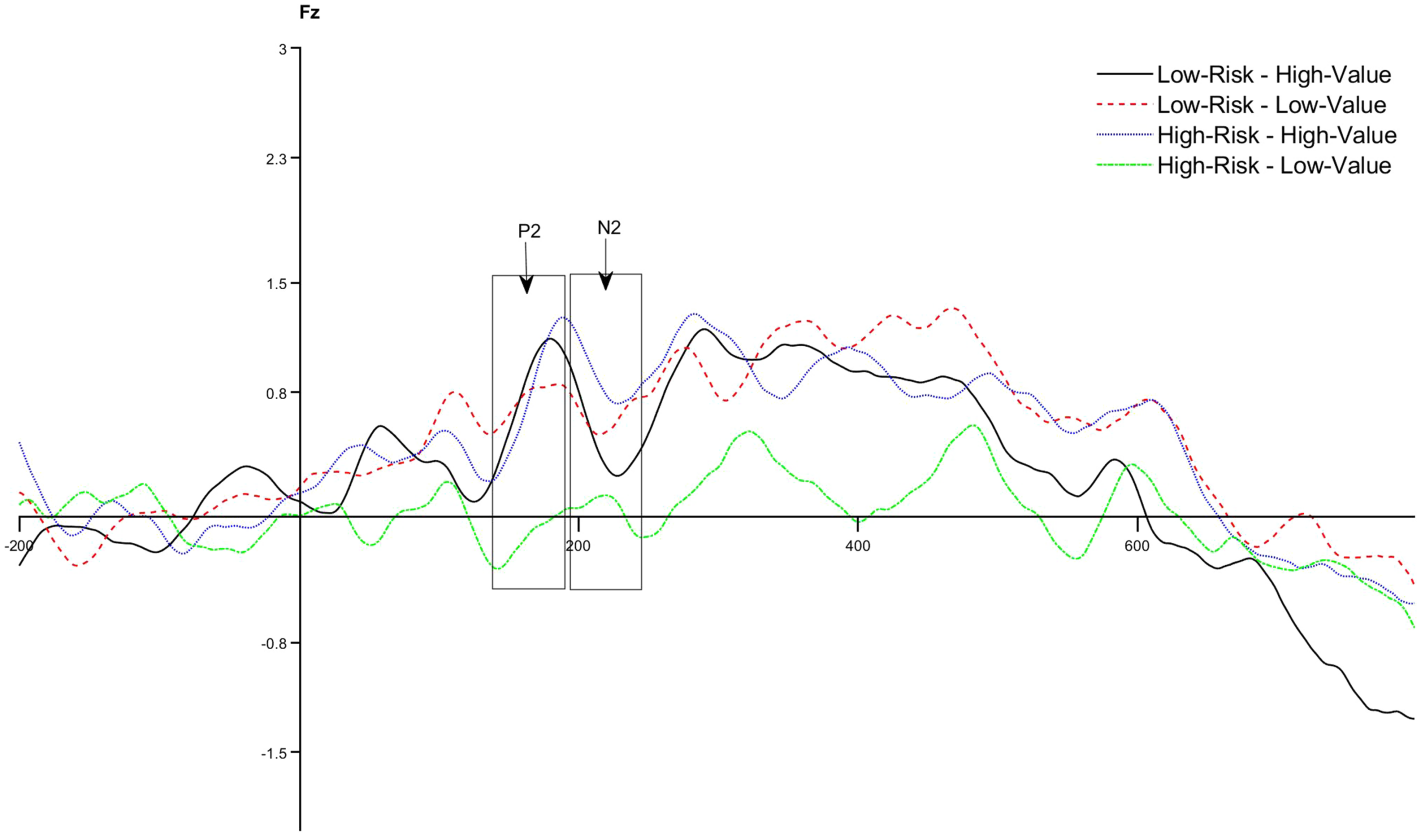
The amplitude of the P2 and N2 component.

A 2 (risks: high-risk vs. low-risk) × 2 (benefits:high-value vs. low-value) × 6 (electrodes: Fz, F1, F2, FCz, FC1, and FC2) repeated measures ANOVA was conducted on the mean amplitude of the P2 component. The results show that the main effect of risk was not significant, with *F* (1,72) = 1.564, *p* = 0.215, *η^2^* = 0.021, indicating that there was no significant difference in attentional resources allocated to different risks; the main effect of benefit is significant, *F* (1,72) = 4.044, *p* = 0.048, *η^2^* = 0.053; the P2 amplitude induced by high value (*M* = 0.738, SD = 1.258) is significantly higher than that induced by low value (*M* = 0.251, SD = 1.312), suggesting that participants allocated more attentional resources to high value services; the main effect of electrode position is not significant, with *F* (5,360) = 0.891, *p* = 0.457, *η^2^* = 0.012. None of the interactions is significant. The amplitude of the P2 component is shown in Figure 3, The ANOVA result for P2 component is shown in Table 3.

**Table 3:** ANOVA results for P2 component.

Dependent variable	Independent variable	*F*	*P*	*η* ^2^
The amplitude of the P2 component	Privacy risk	1.564	0.215	0.021
	Privacy benefit	4.044	0.048	0.053
	Electrode placement	0.891	0.457	0.012

#### N2

A 2 (risks: high-risk vs. low-risk) × 2 (benefits: high-value vs. low-value) × 6 (electrodes: Fz, F1, F2, FCz, FC1, and FC2) repeated measures ANOVA was conducted on the mean amplitude of the N2 component. The results show that the main effect of risk is not significant, with *F* (1,72) = 0.008, *p* = 0.927, *η^2^* < 0.001; the main effect of benefit is not significant, with *F* (1,72) = 0.341, *p* = 0.561, *η^2^* = 0.005; the main effect of electrode position is not significant, with *F* (5,360) = 0.912, *p* = 0.461, *η^2^* = 0.025; the interaction between risk perception and value perception is significant, with *F* (1,72) = 4.540, *p* = 0.037, *η^2^* = 0.059, suggesting that risks and benefits do not affect decision individually, but affect decision together; other interaction effects are not significant.

Simple effect analysis shows that there is no significant difference in N2 amplitude induced by benefit at low risk (*p* = 0.444 > 0.05); the amplitude of N2 induced by benefit at high risk is significantly different (*p* = 0.033 < 0.05); the amplitude of N2 induced by low value (*M* = −0.031, SD = 1.210) is significantly larger than that induced by high value (*M* = 0.623, SD = 1.006), meaning that participants allocated more cognitive resources to low-value services in the high-risk condition, while there was no such difference in the low-risk condition. The amplitude of N2 component is shown in Figure 3, The ANOVA result for N2 component is shown in Table 4.

**Table 4:** ANOVA results for N2 component.

Dependent variable	Independent variable	*F*	*P*	*η* ^2^
The amplitude of the N2 component	Privacy risk	0.008	0.927	0.001
	Privacy benefit	0.341	0.561	0.005
	Electrode placement	0.912	0.461	0.025
	Risk–benefit interaction effect	4.54	0.037	0.059

#### LPP

**Figure 4:**
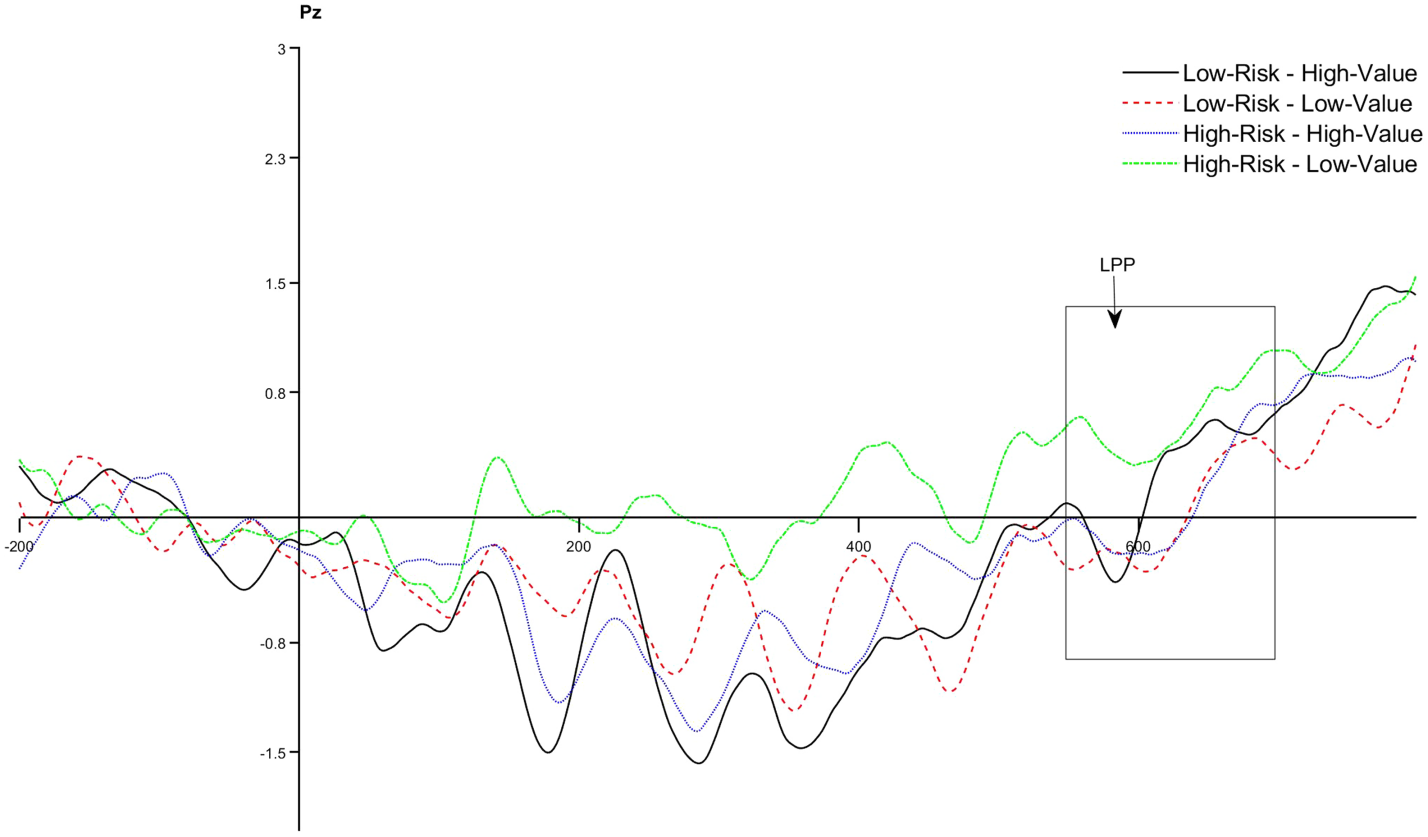
The amplitude of LPP component.

A 2 (risks: high-risk vs. low-risk) × 2 (benefits:high value vs. low value) × 8 (electrodes: Cz, C1, C2, CP1, CP2, Pz, P3, and P4) repeated measures ANOVA was conducted on the mean amplitude of the LPP component. The results show that the main effect of risk perception is not significant, with *F* (1,72) = 0.131, *p* = 0.718, *η^2^* = 0.002; the main effect of value perception is not significant, with *F* (1,72) = 0.987, *p* = 0.324, *η^2^* = 0.014; the main effect of electrode position is not significant, with *F* (7,504) = 2.061, *p* = 0.101, *η^2^* = 0.028. None of the interaction effects is significant, suggesting that risks and benefits can neither affect emotional changes alone nor together. The amplitude of LPP component is shown in Figure 4, The ANOVA result for N2 component is shown in Table 5.

**Table 5:** ANOVA results for LPP component.

Dependent variable	Independent variable	*F*	*P*	*η* ^2^
The amplitude of the LPP component	Privacy risk	0.131	0.718	0.002
	Privacy benefit	0.987	0.324	0.014
	Electrode placement	2.061	0.101	0.028
	Risk–benefit interaction effect	0.337	0.563	0.004

## Discussion

The objective of this study was to investigate the influence of risk and benefit on privacy-related decision-making in the context of personalized services. Privacy calculus theory considers the process of users’ choice of whether to make privacy disclosures as a process of weighing potential risks and benefits, yet there is no uniform conclusion as to how risks and benefits affect privacy decisions. Moreover, we argue that people are both rational and emotional in privacy decisions, and emotions profoundly influence users’ disclosure choices. Therefore, To open up this black box and understand how the factors influencing the privacy decision-making process, ERP was employed in this study to record and analyze the neural activity of participants to better understand the role of risk versus benefit privacy decisions and to explore the influences of emotional changes in privacy decisions.

This study examines how risk and benefit affect the privacy decision-making process in a chronological manner. The EEG results of this study found P2 component, which is related with distribution of attention and the classification of stimulus (Correll et al., 2006; Huang and Luo, 2006), is associated with the benefits of personalized services. These findings are similar to previous studies which argued that the classification of stimuli is reflected in the allocation of different attention resources of the stimulus (Crowley and Colrain, 2004; Doallo et al., 2007; Noguchi and Murota, 2013). In addition, Luck (2014) suggest that the stimulus classification represented by the P2 component is low-level, automatic, and unconscious classification behavior. Therefore, our finding for P2 component indicates that users will focus on the value of personalized services rather than the risk of privacy disclosure when making privacy decisions, and automatically classify personalized services based on value. This supports the prior finding that users are more concerned about the benefits of privacy disclosure than privacy risks and privacy concerns (Lee and Rha, 2016; Zhu et al., 2021).

N2 component, an ERP component reflecting cognitive process, reflects the cognitive conflict and risk perception experienced by individuals (Dennis and Chen, 2007; Yang et al., 2007). In this study, we found neither risk nor benefit could alone affect the N2 amplitude of users, but their interaction did. This result implies that users are in a state of cognitive conflict between risk and benefit when making privacy decisions, which is consistent with Lee and Rha (2016). Interestingly, the conflict between risks and benefits is not entirely different. There is no difference in cognitive conflict induced by high and low benefits when risk is low, but at high risk, low benefit induces greater cognitive conflict. This phenomenon means that when users faced with loss, higher loss will induce greater cognitive conflict, similar to the fact that people are more sensitive to loss proposed by Kahneman and Tversky (2013). Noteworthily, the acceptance of high-risk and high-benefit personalized services, which were allocated fewer cognitive resources, was higher than that of high-risk and low-benefit services, which were allocated more cognitive resources. This finding is similar to the conclusion that the scarcity of cognitive resources will increase the level of privacy disclosure argued by Dennis and Chen (2007) by studying the impact of cognitive load on privacy disclosure. Therefore, we argue that the balance between risk and benefit affect users’ privacy decisions by influencing the allocation of cognitive resources, and the more cognitive resources allocated, the lower the possibility of privacy disclosure.

However, unfortunately, there was no correlation between risk and LPP components from the EEG results. In other words, there was no statistical difference between the emotional valence induced by high risk and that triggered by low risk in the present study. Although we found that participants perceive high risk withholding their privacy, this finding still contradict the results of Mohammed and Tejay (2021) who found that individuals that perceive a high level of privacy risk invoke emotional processing and executive functions which in turn leads to withholding privacy. Loewenstein et al. (2001) proposed a theory, the risk-as-feelings, that highlights the role of emotion experienced at the moment of decision making. They argued that emotional reactions to risky often diverge from cognitive assessments of those risks. Different from Mohammed and Tejay (2021), trust and distrust are not factors involved in this study, because the experimental materials selected in this experiment are all apps that participants are very familiar with. In their study, they found trust was associated the orbitofrontal prefrontal cortex (OFC), which is the brain area involved in emotional regulation and most cognitive processes (Karmarkar and Plassmann, 2019; Pärnamets et al., 2020). In addition, research has shown that other factors, such as personality (Pentina et al., 2016), privacy concerns (Hallam and Zanella, 2017) and types of information (Marwick and Hargittai, 2019), are associated with emotions in privacy decisions. Thus, we believe that risk is not the only factor that influences emotions in the privacy decision-making process, and there are other factors that affect emotions in the process of privacy decision-making.

Although this study has found how risks and benefits affect privacy decision in chronological order, there are still some deficiencies. Firstly, as mentioned above, there are many factors not involved in this study, such as trust, privacy concerns, etc. Secondly, the experimental material selected in this study are all apps that participants are familiar with. One previous study have shown that consumers will make decision quickly based on their existing preferences and experience when face with familiar products, which is manifested as activation of dorsolateral prefrontal cortex (dlPFC) (Pärnamets et al., 2020). When faced with an unfamiliar app, the user’s privacy decision may be different from that of the familiar app. Therefore, future studies can start from the following aspects: 1. More factors are introduced in future experiments to study the privacy decision process; 2. Focus on the differences in privacy decisions between familiar apps and unfamiliar apps.

## Contribute and implication

This study provides both theoretical and methodological contributions, as well as Management implications. It contributes to the privacy paradox field primarily by addressing the gap in literature by investigating the process of privacy decision-making. Previous studies considered people as an indivisible whole, assumed that users are a black box, and studied the causes of users’ privacy-related decisions through self-reported methods such as questionnaires and interviews. This study took an objective and exploratory approach to decompose the process of privacy decision-making, based on the findings of neuroscience and information privacy literature, and proposes a novel explanation for the causes of privacy decision. Moreover, we found, by comparing our experiments with those of previous studies, that risk is not the only factor that can affect emotional changes in privacy decisions, and emotional changes are influenced by a combination of multiple factors. This finding could help further research into the process of privacy decision-making.

As discussed by previous studies, self-reported data may often involve biases or inaccurate answers (Dimoka, 2011; Fu et al., 2022). Different from self-reported, we can obtain the real thoughts of participants through proper analysis neural activity of them, which contains all of their inner opinions. Further, experimentation manipulates the conditions under which factors operate, as well as, observe real behavior, thereby providing a more explicit explanation (Mohammed and Tejay, 2021).

For enterprises providing personalized services, how to obtain more user information is a necessary condition to improve the accuracy of personalized recommendation, which is the premise to increase user engagement. Our findings provide some suggestions for enterprises to improve user acceptance of personalized services. We found that users subliminally classify personalized services through benefits, which affect users’ acceptance of personalized services. Studies have shown that both the interaction mode between app and users and the app interface itself can affect users’ value perception (Li et al., 2017; Veltri and Ivchenko, 2017). Therefore, we argue enterprises can improve acceptance from these two aspects. In addition, users will invest more cognitive resources to personalized services when they perceive high privacy risks, resulting in lower degree of privacy disclosure. One research shows that users’ control of privacy disclosure content can reduce the perception of privacy risk (Li et al., 2017), and thus increase the degree of privacy disclosure.

## Conclusion and limitation

This study investigated the influence of risk and benefit on the process of privacy decision-making through ERP. Our findings are: Users subconsciously categorize personalized services based on benefit; Privacy calculus affects privacy decision by influencing the allocation of cognitive resources for personalized service, and the scarcity of cognitive resources increases the degree of privacy disclosure; Emotional change in privacy decision is the result of many factors, not the result of privacy risk alone. The contribution of our study is the use of a neuroscientific approach to provide a new explanatory perspective on privacy paradox research and to provide some suggestions for companies to increase user privacy disclosure. The summary of hypotheses results are shown in Table 6.

**Table 6:** Summary of hypotheses results.

Hypothesis	Result	
H1	High value services → larger P2 amplitude	Supported
H2	interaction of risk and benefit → the amplitude of N2 component.	Supported
H3	high risk can induce higher LPP amplitude	Not supported

This study was subject to some limitations. Firstly, due to time effort and funding limitations, the sample size was selected small and the subjects were homogeneous in age and occupation, although the sample size was consistent with most EEG studies and future studies could expand the sample size as conditions allow. Secondly, this study focused on the effects of risk and benefit on privacy decisions, but there are other influences that were not included in the study, and these are areas that could be studied in depth in the future. Thirdly, the stimulus materials used in the experiment were all well-known apps, and how the participants responded when faced with unfamiliar apps was not addressed in this study, future research could add more factors and add stimulus material unfamiliar to the participants to the experiment, and continue to delve into the causes of the privacy paradox. Fourthly, functional brain imaging studies can confirm that certain brain regions are associated with certain decision-making behaviors but this does not exclude that certain other brain regions also perform the corresponding decision-making tasks, and this limitation in the research tools may be solved in the future when more advanced tools are available.

## Data availability statement

The original contributions presented in the study are included in the article/supplementary material, further inquiries can be directed to the corresponding author.

## Ethics statement

Ethical review and approval was not required for the study on human participants in accordance with the local legislation and institutional requirements. The patients/participants provided their written informed consent to participate in this study.

## Author contributions

JF and XL designed the research. JZ and XL performed the whole ERP experiment and the data analysis. JZ wrote the whole manuscript. All authors have read and approved the final manuscript.

## Funding

National Social Science Foundation of China (21CJL006); Key Project of Soft Science Research in Henan Province (222400410004); Major Project of Applied Research on Philosophy and Social Science in Henan Higher Education Institutions (2023-YYZD-28).

## Conflict of interest

The authors declare that the research was conducted in the absence of any commercial or financial relationships that could be construed as a potential conflict of interest.

## Publisher’s note

All claims expressed in this article are solely those of the authors and do not necessarily represent those of their affiliated organizations, or those of the publisher, the editors and the reviewers. Any product that may be evaluated in this article, or claim that may be made by its manufacturer, is not guaranteed or endorsed by the publisher.
